# Non-covalent polymer wrapping of carbon nanotubes and the role of wrapped polymers as functional dispersants

**DOI:** 10.1088/1468-6996/16/2/024802

**Published:** 2015-03-10

**Authors:** Tsuyohiko Fujigaya, Naotoshi Nakashima

**Affiliations:** 1 Department of Applied Chemistry, Graduate School of Engineering, Kyushu University, 744 Motooka, Fukuoka, 819-0395, Japan; 2 The World Premier International Research Center Initiative, International Institute for Carbon-Neutral Energy Research (WPI-I2CNER), Kyushu University 744 Motooka, Fukuoka, 819-0395, Japan; 3 JST-CREST, 5 Sanbancho, Chiyoda-ku, Tokyo, 102-0075, Japan

**Keywords:** carbon nanotubes, dispersant, non-covalent functionalization, polymer wrapping

## Abstract

Carbon nanotubes (CNTs) have been recognized as a promising material in a wide range of applications from biotechnology to energy-related devices. However, the poor solubility in aqueous and organic solvents hindered the applications of CNTs. As studies have progressed, the methodology for CNT dispersion was established. In this methodology, the key issue is to covalently or non-covalently functionalize the surfaces of the CNTs with a dispersant. Among the various types of dispersions, polymer wrapping through non-covalent interactions is attractive in terms of the stability and homogeneity of the functionalization. Recently, by taking advantage of their stability, the wrapped-polymers have been utilized to support and/or reinforce the unique functionality of the CNTs, leading to the development of high-performance devices. In this review, various polymer wrapping approaches, together with the applications of the polymer-wrapped CNTs, are summarized.

## 1. Introduction

CNTs are cylindrical graphene tubes with a one-dimensional *π*-conjugated structure [1]. Due to the outstanding electrical, mechanical, thermal and optical properties, CNTs are promising candidates for various applications such as electronics, sensors, energy conversion and storage devices [2]. During the early stage of CNT research, the limited solubility of CNTs, due to their high aspect ratios and strong van der Waals interactions, hindered the development of CNT applications [3]. Only a limited number of organic solvents, such as *o*-dichlorobenzene (ODCB), *N*-methylpyrrolidinone (NMP), *N,N*-dimethylformamide (DMF) and *N,N*-dimethylacetamide (DMAc), are known to disperse CNTs to some extent, but the degree of isolation and the stability are not sufficient for many of the applications [4–12].

**Figure 1.**
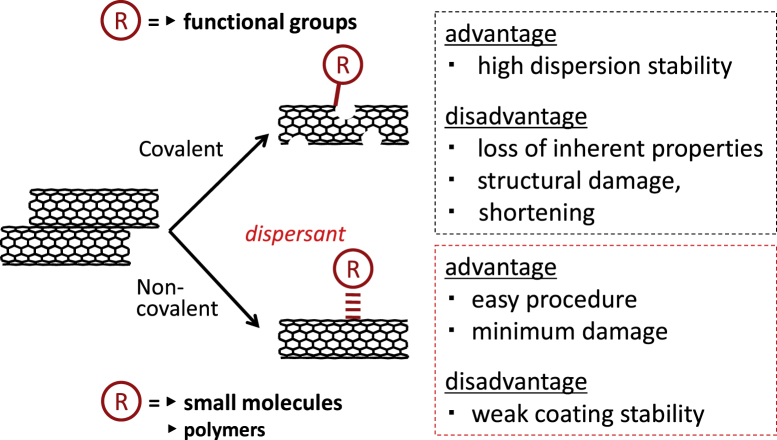
Main CNT functionalization methods.

Based on the results of the significant amount of research, the methodology to exfoliate the bundled structures of the CNTs and disperse them in solvents has been established in which the engineering of the surface of the CNTs using small molecules or polymers in a covalent or non-covalent way is the major strategy (figure 1) [13–15].

For the covalent modifications, the oxidation of the surface to introduce the carboxyl groups [16–24] and the chemical reactions to connect the functional moieties are typical approaches. Generally speaking, the covalent modifications are superior to the non-covalent modification in terms of the stability of the functionalization. In particular, the covalent approach easily leads to the effective reinforcement of the polymer films due to the effective load transfer from the polymer matrix to the CNTs through the covalent bonding [25–30]. However, the covalent modification changes the intrinsic properties of the CNTs, such as conductivity and mechanical toughness, and often cuts the CNTs into shorter tubes [31]; thus, the non-covalent modification is superior in most cases in order to utilize the inherent properties of the CNTs [14, 15, 30, 32–39]. In addition, non-covalent modifications are characterized as their simple procedure, typically just by the mixing of CNTs with molecules under a shear force treatment such as sonication. In this review, the molecules used for the non-covalent modification of CNTs are called ‘dispersants’.

**Figure 2.**
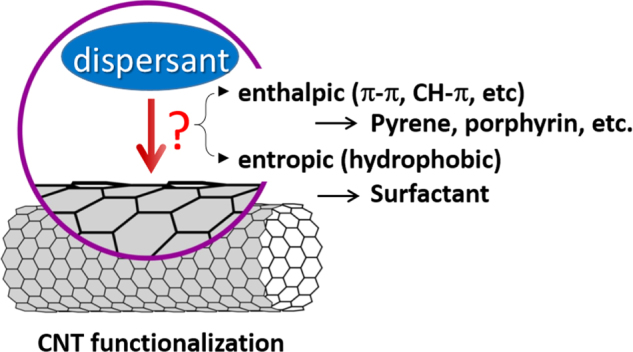
Dispersant-CNT interactions.

**Figure 3.**
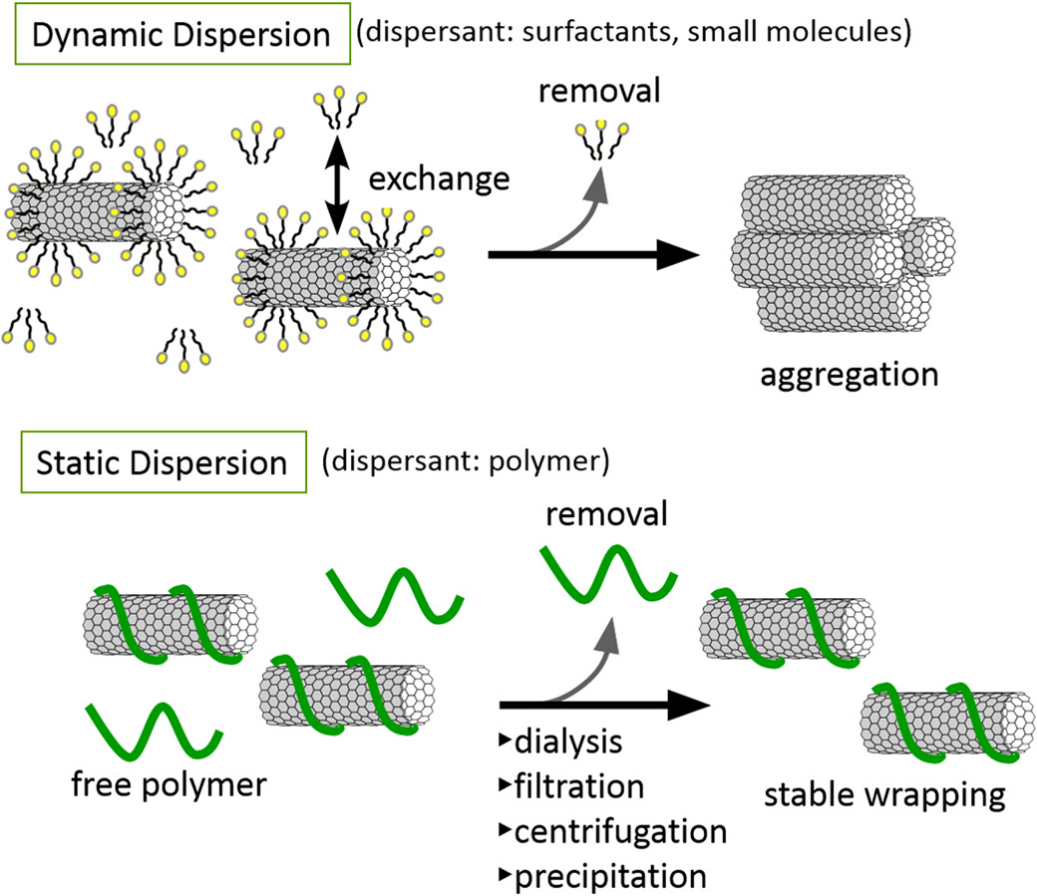
Schematic illustrations for the dynamic (upper) and static (lower) dispersion of CNTs.

Non-covalent functionalization is realized via 1) enthalpy-driven interactions, such as *π*−*π*, CH−*π*, NH−*π*, etc, between the CNT surface and the dispersants and/or 2) entropy-driven interaction; i.e. hydrophobic interaction using surfactants (figure 2) [40]. In the case of the surfactant dispersion, sodium dodecyl sulfate (SDS) [41–43], sodium dodecylbenzene sulfonate (SDBS) [44–48], sodium cholate (SC) [49–51], cethyltrimethylammonium bromide (CTAB) [46, 52], Brij [46, 51], Tween [46, 51] and Triton X [44, 46, 51, 53] have typically been used due to their availability and cost [50]. It is important to understand that the surfactant molecules on the surface of the CNTs are in a dynamic equilibrium between the surfactants in the bulk solution (figure 3, upper) [54]. Therefore, the dispersants are easily removed by filtration or dialysis, resulting in the aggregation of the CNTs (figure 3) [55]. Similar dynamic dispersions also take place in the case of the enthalpy-driven functionalization using small molecules as the dispersant. On the other hand, when the polymer is used in the enthalpy-driven functionalization, static dispersion due to the multi-point interaction between polymers and the surface of the CNTs is often realized [55]. In this case, the non-covalently surrounded polymers remained even after the washing process, such as filtration, to provide the ‘polymer-wrapped CNTs’. In some applications, such wrapped dispersants act as a contaminate, but in some cases, the wrapped CNTs synergistically improve the performance of the CNTs if the polymers are strategically designed. In this review, such polymers that offer additional functions to the CNTs are categorized as ‘functional dispersants’. Due to the tailorable design of the polymers, the concept of the functional dispersant has recently been widely recognized and utilized.

In this review, some examples of the non-covalent functionalization of CNTs based on the polymer wrapping approach and the roles of the functional dispersants are summarized.

## 2. Type of polymer for wrapping

**Figure 4.**
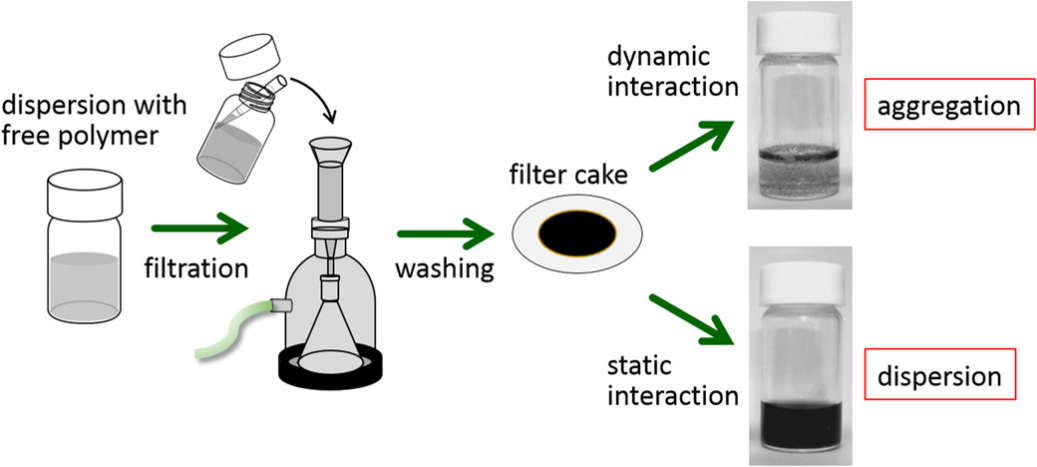
Schematic procedure of the filtration process.

For the polymer wrapping of CNTs, various interactions, including *π*−*π*, CH−*π* and cation−*π*, function to adsorb on the surfaces of the CNTs. In this chapter, the types of polymers used for the wrapping are summarized based on their structural features. One of the advantages of the polymer wrapping is the thermodynamically stable coating on the surface of the CNTs, and it is possible to remove the unbound (free) polymer while leaving the wrapping polymer on the CNT surface (figure 3, lower). Removal of the unbound polymer in the bulk solution can be carried out by (1) dialysis [56, 57], (2) a precipitation/decantation cycle [58], (3) a filtration/washing process [59–61], (4) a ultracentrifugation/decantation process [58, 62] and (5) a chromatographic separation [57]. In the case of the filtration process, it is quite simple to distinguish the stable dispersion since the nice dispersion is achieved again from the washed materials when the stable wrapping has taken place (figure 4). Note that not all the papers detail the presence of the unbound polymer in the solution, and it is highly recommended to recognize the effect of the unbound polymer even when the authors did not describe it since such polymers sometimes affect displayed data.

### 2.1. *π*-conjugated polymers

**Figure 5.**
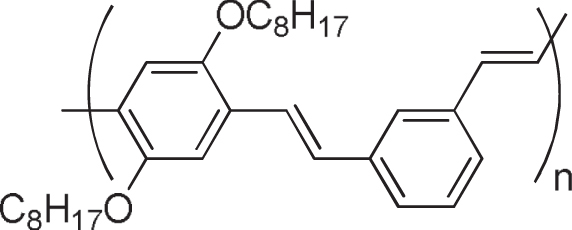
Chemical structure of PmPV.

**Figure 6.**
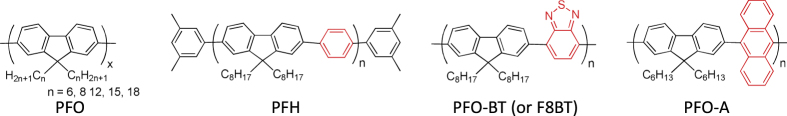
PFO and its derivatives that dissolve s-SWCNTs reported from other groups.

It is reasonable to use *π*-conjugated polymers for the wrapping of the *π*-conjugated surfaces of the CNTs in which effective interactions, such as *π*−*π* and/or CH−*π*, are expected. Pioneering studies of dispersing CNTs by a *π*-conjugated polymer into solvents were carried out using poly(*p*-phenylenevinylene) derivatives (PPVs) as the polymer dispersant [63–65]. The CNTs formed a stable dispersion in the organic solution of PPVs, suggesting the formation of the polymer-wrapped CNTs. In addition to the dispersion of CNTs [66–82], the PPV wrapping was also utilized for the extraction of single-walled carbon nanotubes (SWCNTs) with specific chiral indices. Keogh *et al* and Coleman *et al* used the poly(*m*-phenylene-co-2,5-dioctoxy-*p*-phenylenevinylene) (PmPV, figure 5) to preferentially disperse SWCNTs with specific chiral indices, leaving the others in the precipitate [83–86]. Although a detailed mechanism of the selective dispersion is still unknown, it is assumed that the *π*-conjugated polymers with a rigid backbone exhibit the selectivity for the specific chiral indices by aligning their backbones along the SWCNTs’ surfaces with a preferential angle in order to maximize the interaction on the *π*-surface [87]. Selective extraction of semiconducting SWCNTs (s-SWCNTs) by *π*-conjugated polyfluorene (PFO) and their derivatives has been reported by Nish *et al* in which poly(9,9-dioctylfluorenyl-2,7-diyl) (PFO) and poly[(9,9-dioctylfluorenyl-2,7-diyl)-*alt*-*co*-(1,4-benzo-2,1,3-thiadiazole)] (PFO-BT or P8BT) (figure 6) were used [88].

**Figure 7.**
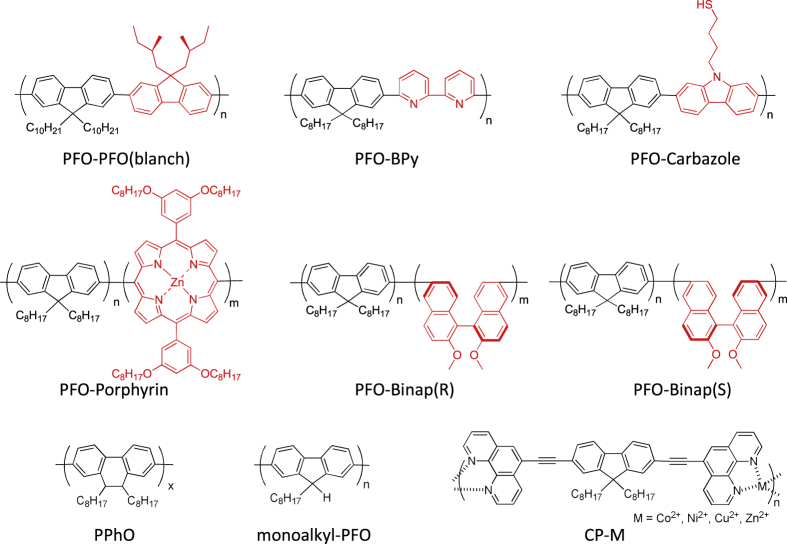
PFO derivatives and the analogs selectively disperse s-SWCNTs reported from our group.

**Figure 8.**
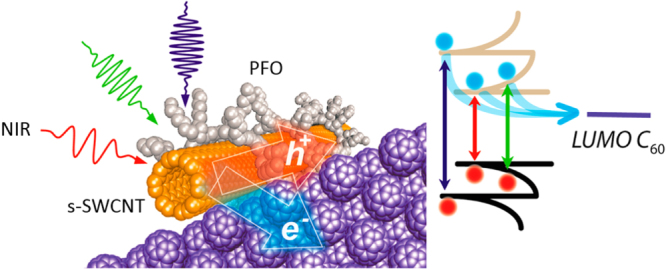
(left) Schematic depicting charge transfer at the s-SWCNT/C_60_ interface under NIR irradiation. (right) Energy diagram of s-SWCNTs and C_60_, where LUMO stands for lowest unoccupied molecular orbital. Reproduced with permission from D J Bindl and M S Arnold 2013 *J. Phys. Chem.* C **117** 2390. Copyright 2013 American Chemical Society.

**Figure 9.**
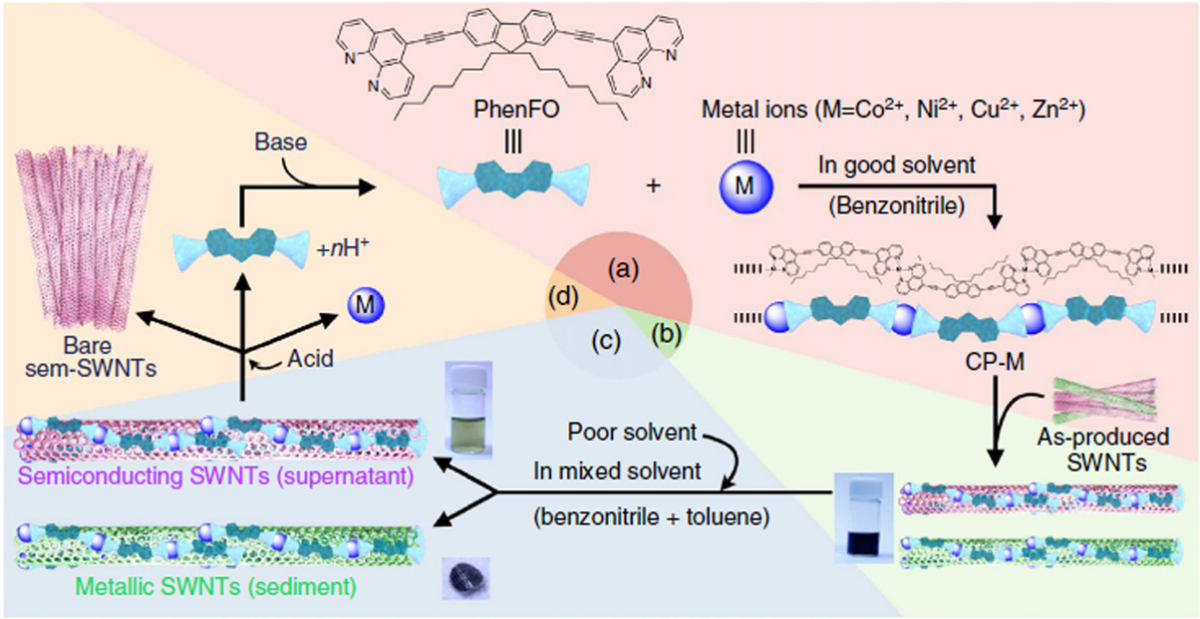
A method for s-SWCNT sorting using removable dispersant. The purification method starts from (a) the preparation of CP-M (M = Co, Ni, Cu and Zn), (b) dispersion of as-produced SWCNTs, (c) separation of s- and m-SWCNTs and (d) removal and recovery of the adsorbents. Chemical structure of PhenFO is shown in step (a). Reprinted by permission from Macmillan Publishers Ltd: F Toshimitsu and N Nakashima 2014 *Nat. Commun.* **5** 5041, copyright 2014.

Compared to the other separation techniques, such as gel chromatography [89–93] and density gradient ultracentrifugion (DGU) [94, 95], PFO-based extraction of s-SWCNTs is quite attractive due to its simplicity as well as its high purity [96]. Very importantly, the tailorable design of the PFO allowed researchers to develop a wide variety of PFO-based copolymers for the separation of the SWCNTs [97]. Indeed, we demonstrated that many monomers are incorporated into the PFO for the s-SWCNT extraction (figure 7) [98–105]. Thanks to the high purity of the obtained s-SWCNTs, the extracted s-SWCNTs are considered as a promising material for next-generation SWCNT-based semiconducting devices. Izard *et al* reported an on/off ratio greater than 10^5^ in a field effect transistor (FET) fabricated from the s-SWCNT network extracted by PFO wrapping, while the unsorted SWCNTs gave only 10^2^ ∼ 10^3^ [61]. In 2010, Bindl *et al* reported light harvesting in the near-IR (NIR) region using PFO-sorted s-SWCNTs as the chromophore, where C_60_ was incorporated as the acceptor (figure 8) [106–108]. In these examples, due to the high-quality separation of the s-SWCNTs by PFO, a significant enhancement of the efficiency was obtained compared to the device fabricated with unsorted SWCNTs. However, in these applications, the wrapped PFOs were used only for the separation but remained wrapped in the devices. It was pointed out that the organic residual remaining on the surface of CNTs often diminishes the performance of the devices, especially for semiconducting applications [94], and in such cases the complete removal of the wrapped polymers is required. For instance, Bisri *et al* developed a two-step ultracentrifugation method to remove the polymer from the polymer-dispersed CNT solution [62]. In this method, they obtained a polymer less than 7 mg mL^−1^, but wrapping of the polymer was so tight that the perfect removal of the polymer was not possible. Recently, we have designed the fluorene monomer carrying two ligand units (PhenFO), which polymerize in the presence of metal ions to give a PFO derivative (CP-M) by coordination (figure 7). In this system, the wrapped PFO was easily removed by the addition of an acid to decompose the coordination after the extraction of the s-SWCNTs (figure 9) [109]. As the result, complete removal of the polymer was realized.

**Figure 10.**
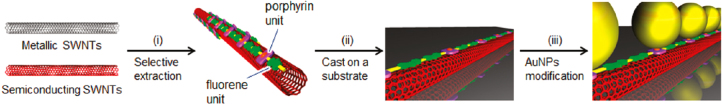
Schematic illustration of the separation of s-SWCNTs using PFO-porphyrin and an attachment of gold nanoparticles (AuNPs). Reprinted with permission from H Ozawa *et al* 2011 *J. Am. Chem. Soc.* **133** 14771. Copyright 2011 American Chemical Society.

In contrast to such an approach, it is not necessary to remove the wrapped polymer in the concept of ‘functional dispersant’. In order to utilize the wrapping PFO as the functional dispersant, we developed a PFO-based dispersant by introducing a carbazole-based co-monomer bearing a thiol group (PFO-carbazole, figure 7). Since the thiol group binds to the Ag surface, the PFO-wrapped s-SWCNTs were decorated with the Ag nanoparticles after the separation [103]. A similar decoration with Au nanoparticles was also achieved using the s-SWCNTs wrapped with a porphyrin-containing PFO copolymer (PFO-porphyrin, figures 7 and 10) [102]. In these examples, the wrapped PFO were functioned for the anchoring of metal nanoparticles.

**Figure 11.**
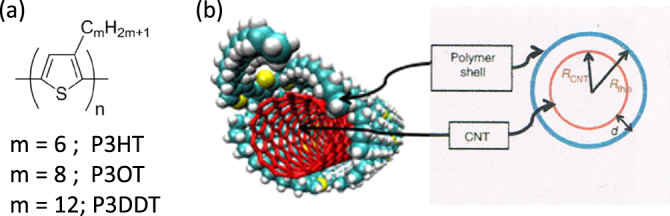
(a) Chemical structure of poly(3-alkylthiophenes). (b) Schematics of a cross-sectional geometrical view of the polymer–SWCNT supramolecular structure. Reprinted by permission from Macmillan Publishers Ltd: H W Lee *et al* 2011 *Nat. Commun.* **2** 541, copyright 2011.

Poly(3-alkylthiophenes), figure 11(a), such as poly(3-hexylthiophene) (P3HT), are also known to wrap CNTs and are used mostly for organic photovoltaic applications [110]. It was pointed out that not only the *π*−*π* interaction but also the sulfur atom in the backbone plays an important role for the adhesion based on MD calculations [111]. Goutam *et al* found that P3HT rapidly degrades in organic solvents containing dissolved molecular oxygen when irradiated with an UV light, but that was not the case for the P3HT-wrapped CNTs [112]. It was suggested that the *π*–*π* interaction between the P3HT-CNT composite improves the stability of the *π*-conjugation system, thereby preventing photosensitization and a reduced opportunity for the reaction of singlet oxygen with the P3HT. This enhanced stability explained the higher stability and efficiency of the P3HT/CNT devices compared with the devices composed from P3HT [113]. In this example, the function of the polymer was reinforced by the incorporation of CNTs. In 2011, Lee *et al* found that regioregular poly(3-alkylthiophenes) selectively disperse s-SWCNTs by a polymer wrapping mechanism (figure 11(b)) [114]. Using this technique, they fabricated high-performance transistors formed with a s-SWCNT network and observed a charge-carrier mobility as high as 12 cm^2^ V^−1^ s^−1^ and an on/off ratio of >10^6^.

Other *π*-conjugated polymers, such as polypyrrole (PPy) and polyaniline (PANI) [115], as well as their derivatives also wrap the CNTs, and many such studies are summarized in an excellent review paper [115]. In particular, for these polymers, electropolymerization using CNTs as the electrode enables homogeneous wrapping by the polymers. It is quite unique since, in this approach, stable wrapping was achieved even in the absence of the strong interaction between the wrapping polymer and CNT surfaces.

### 2.2. Aromatic polymers

**Figure 12.**
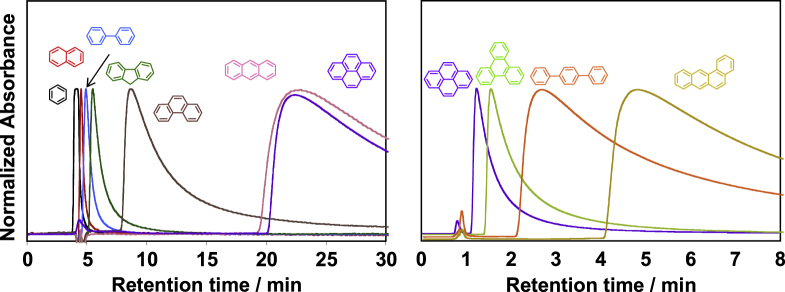
Chromatogram of the series of aromatic compounds analyzed using CNT as the stationary phase. Chromatograms of (black) benzene, (red) naphthalene, (light blue) biphenyl, (green) fluorene, (brown) phenanthrene, (pink) anthracene, (purple) pyrene, (light green) triphenylene, (orange) *p*-terphenyl and (yellow) tetraphene obtained from the SWCNT-column; eluent: tetrahydrofuran, flow rate 0.1 mL min^−1^ (left) and 0.5 mL min^−1^ (right).

Aromatic condensation polymers having an aromatic system in the main-chain, such as polyimides (PIs), are the polymers that can wrap the CNTs [116]. Our chromatography studies using CNTs as the stationary phase revealed that the one-dimensional aromatic compounds, such as tetraphene, exhibited a stronger affinity with the CNT surfaces than the other analogs with the same number of the aromatic rings, such as pyrene or triphenylene, due to the effective overwrapping with the one-dimensional CNT surfaces (figure 12) [117, 118].

**Figure 13.**
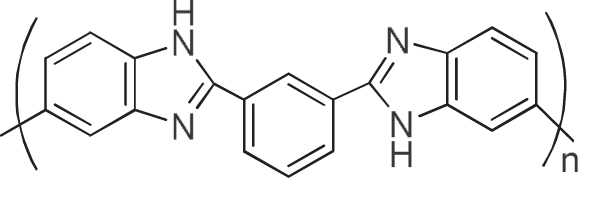
Chemical structure of PBI.

We considered that the results well explained the effective interaction of the aromatic condensation polymers, such as PIs, with the CNTs. By taking advantage of the remarkable thermal stability of these polymers and CNTs, the composites are fascinating for the use under harsh conditions such as aerospace applications. We have reported that a PI having a sulfonic acid salt (PI-SO_3_Na) effectively exfoliated and dispersed SWCNTs in organic solvents for long periods of time [116]. It is interesting to note that the concentration of SWCNTs in a dimethyl sulfoxide (DMSO) solution of the PI-SO_3_Na reached as high as 2–3 mg mL^−1^, and the mixtures formed physical gels above 1.8 mg mL^−1^. Polybenzimidazoles (PBIs, figure 13) also individually and effectively dispersed both SWCNTs [119] and multi-walled carbon nanotubes (MWCNTs) [59] in organic solvents, such as DMAc and NMP, via a polymer wrapping mechanism. PBI-wrapped CNTs were obtained after filtering the dispersion and successive washing with DMAc to remove the unbound PBIs in the solution.

Wrapping of CNTs by PBIs was so effective that the re-dispersion of the wrapped CNTs was possible (figure 4). When using PBI, the wrapping thickness was estimated to be ∼1 nm by dividing the total volume of the PBI by the surface area of the CNTs [59, 60]. Similar stable wrapping was confirmed in polyamide [120].

### 2.3. Non-aromatic polymers

Non-aromatic polymers, such as commercially available poly(vinyl alcohol) [121] and poly(vinylpyrrolidone) [122], have been reported to disperse CNTs via the wrapping mechanism. Baskaran *et al* successfully prepared a stable dispersion of MWCNTs in organic solvents with the aid of polybutadiene, polyisoprene, poly(methyl methacrylate) (PMMA) or poly(ethylene oxide) [123]. They pointed out the importance of the CH–*π* interaction for the dispersion of these non-aromatic polymers. Biological molecules, including peptides [124–129] and proteins [130–132], are also important CNT dispersants in which the hydrophobic domains play a significant role in the interaction [132]. Other biopolymers, such as chitosan [133] and gelatin [134], also wrap the CNTs and assist their dispersion. Significant examples of the wrapping by biological polymers are the helical wrapping of CNTs by amylose [135, 136] and *β*-1,3-glucans [137, 138] to render the hybrid water dispersible in which the multi-point OH−*π* interaction is considered to contribute to the wrapping. Among the biopolymer dispersants, carboxymethyl cellulose sodium salt (CMC) is known to be one of the most effective dispersants for CNTs, and stable individual isolation of SWCNTs was achieved in high concentrations [139]. We utilized the stable isolation of SWCNTs by CMC for *in situ* spectroelectrochemical studies of the SWCNTs in which a stable photoluminescence (PL) of SWCNTs is necessary upon potential cycling [140–143].

In addition, these biopolymer-wrapped CNTs have often been used for the application of cell or tissue culturing since the fibrous 2D or 3D network structure formed by the CNTs resembles native extracellular matrices. In these applications, polymer wrapping using chitosan, gelatin, collagen and CMC served to weaken the potential toxicity and to reinforce the mechanical toughness of the scaffold [144–147].

**Figure 14.**
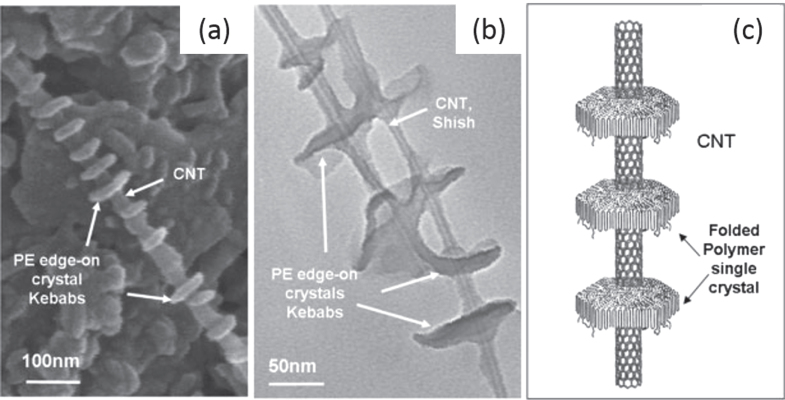
(a) SEM image of MWCNTs decorated by disc-shaped PE single crystals. (b) TEM image of enlarged PE/MWCNT shish kebab structure. (c) Schematic representation of the PE/CNT shish kebab structure. The PE forms folded lamellar single crystals on the CNT surface with polymer chains perpendicular to the lamellae. Reproduced with permission from C Y Li *et al* *Adv. Mater.* **17** 1198. Copyright 2005 John Wiley and Sons.

Another interesting example was reported by Naito *et al* [148]. They developed poly(dialkylsilane)-wrapped SWCNTs through a strong CH−*π* interaction between the alkyl side chains and the SWCNT surfaces. In another example, the CH–*π* interaction between polyethylene (PE) and highly ordered graphitic surfaces of the CNTs induced the crystallization PE [149]. As a result, formation of the ordered shish kebab structure was observed (figure 14) [150, 151].

### 2.4. Cationic polymers

Cationic polymers, such as poly(diallyldimethylammonium chloride) (PDDA) [152, 153], poly(allylamine hydrochloride) [154] and PANI, were often used for the non-covalent CNT wrapping. For PDDA, Yang *et al* pointed out the role of the *π*−*π* interaction [152]. They excluded a possible electrostatic interaction between the positively charged PDDA and COO^−^ groups of the CNTs by using unoxidized CNTs (oxygen concentration <3%). Generally speaking, we need to recognize that pristine CNTs are not always free from the oxygen functional groups, and it is rather hard to deny the possibility of the ionic interaction between the positively charged polymer and the pristine CNTs. Some commercially available CNTs are covered with amorphous carbon that contains 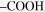 groups. Therefore, characterizing and reporting the purity of CNTs is crucial for many experiments.

### 2.5. Block polymers

**Figure 15.**
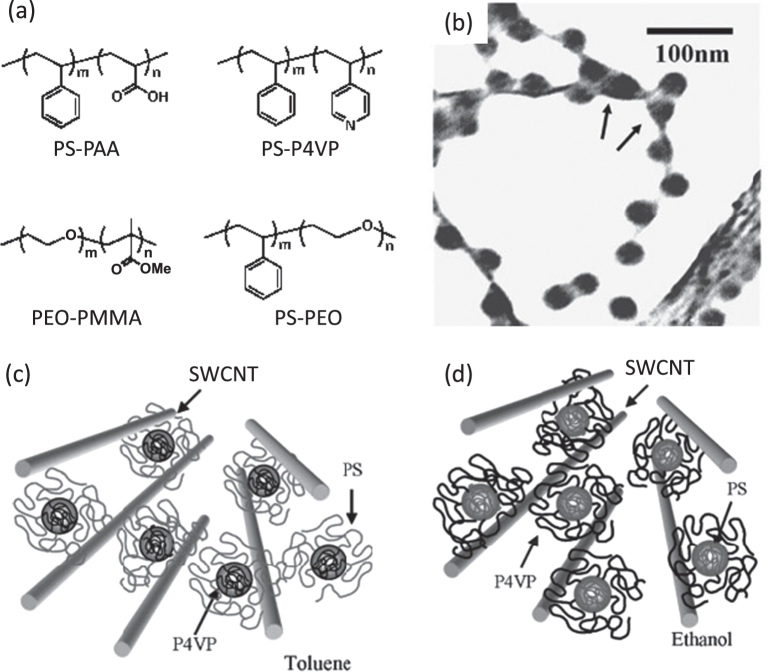
(a) Examples of the chemical structures of the block copolymer-based dispersants. (b) TEM bright-field image of SWCNTs dispersed with PS-P4VP. Micelles are located between two nanotubes, by indicated arrows, implying a possible de-bundling of SWCNTs by micelles. (c), (d) Schematic model of the nanostructure of SWCNTs and block copolymer. PS and P4VP are selectively adsorbed on the surface of nanotubes in (c) toluene and (d) ethanol, respectively. Parts (b)–(d) reproduced with permission from H-i Shin *et al* 2005 *Macromol. Rapid Commun.* **26** 1451. Copyright 2005 John Wiley and Sons.

It is also believed that amphiphilicity of the polymers may contribute to the dispersion of the CNTs through a micelle-encapsulation mechanism. Kang and Taton found that the micelle formation in a DMF solution of polystyrene-*b*-poly(acrylic acid) (PS-PAA, figure 15) [155] induced by water addition enabled the dispersion of SWCNTs. A wide range of block copolymers were reported to disperse CNTs through the micelle encapsulation mechanism in which either polystyrene (PS) [155–163] or polyethylene oxide (PEO) [158, 159, 164, 165] units were introduced as the blocks in most of the cases (figure 15(a)). Shin *et al* found by TEM analysis that in the case of a PS-*b*-poly(4-vinyl pyridine) (P4VP) block copolymer as the dispersant, PS domains were exposed to the outer surfaces in a non-polar toluene solution, while a P4VP domain was located to an outer surface in the polar ethanol solution (figures 15(b)–(d)) [161]. This observation provided a strong indication of the mechanism of the micelle encapsulation of CNTs using the block copolymers.

### 2.6. Pendant polymers

Even if the backbone of the polymers only possess a weak interaction on the surfaces of the CNTs, the tailorable design of the polymers enables stable wrapping by incorporating the pendant moieties having a strong affinity to the CNT surfaces in the polymer side chains. This concept was pioneered by Petrov *et al* using pyrene as the pendant moiety [166]. In this approach, polycyclic aromatic moieties, such as pyrene [167–191] and porphyrin [192], were often introduced [166, 193–197] since they have been proved to show an effective non-covalent interaction with the CNT surfaces [167–191, 198]. As a matter of fact, we revealed that the pyrene moiety acted as a more efficient functional group compared to the naphthyl and phenyl groups for the dispersion [167]. While the monomeric dispersants having a pyrene moiety precipitated CNTs upon heating at around 50 °C, we found that the polymers containing pyrenes in the side chain showed a higher stability and no precipitation up to 95 °C [194]. This result clearly indicates the advantage of a polymer-based dispersant when compared to the monomeric one.

**Figure 16.**
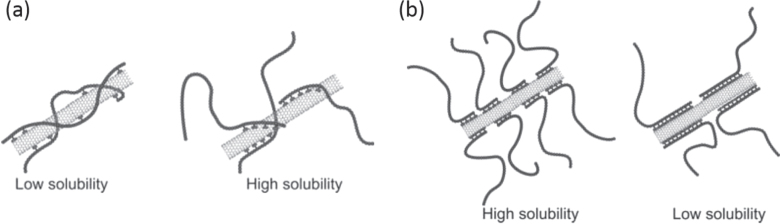
Schematic diagram of nanotube interactions with (a) a pyrene-containing random copolymer (left) versus a block copolymer (right) and (b) short pyrene-functionalized blocks (left) and long pyrene-functionalized blocks (right) in block copolymers. Reproduced with permission from G J Bahun *et al* 2006 *J. Polym. Sci., A: Polym. Chem* **44** 1941. Copyright 2006 John Wiley and Sons.

As for the effective design of the pendant-type dispersants, in 2006, Bahun *et al* reported the effect of the pendant sequence between the random versus block and found a limited solubility for the randomly labeled polymers, while a higher solubility was observed for the block copolymer with one pyrene block (figure 16(a)) [196]. The result was reasonable because for the block copolymer, the other domain can be free to extend into the solution to achieve high solvation. They also revealed that a much longer pyrene block resulted in a decrease in solubility (figure 16(b)) [196]. In 2008, it was reported that only one pyrene unit in the end of the polymer chain was long enough to disperse CNTs [199–201]. Essentially, a multipoint interaction is required for the formation of a stable polymer wrapping, and a more detailed study in terms of the wrapping stability is required in this approach.

Polypeptides with aromatic side chains were also used to disperse CNTs through the *π*−*π* interactions. In this regard, polypeptides containing tryptophan [202] and phenylalanine [203] are known to provide favorable stabilizations.

**Figure 17.**
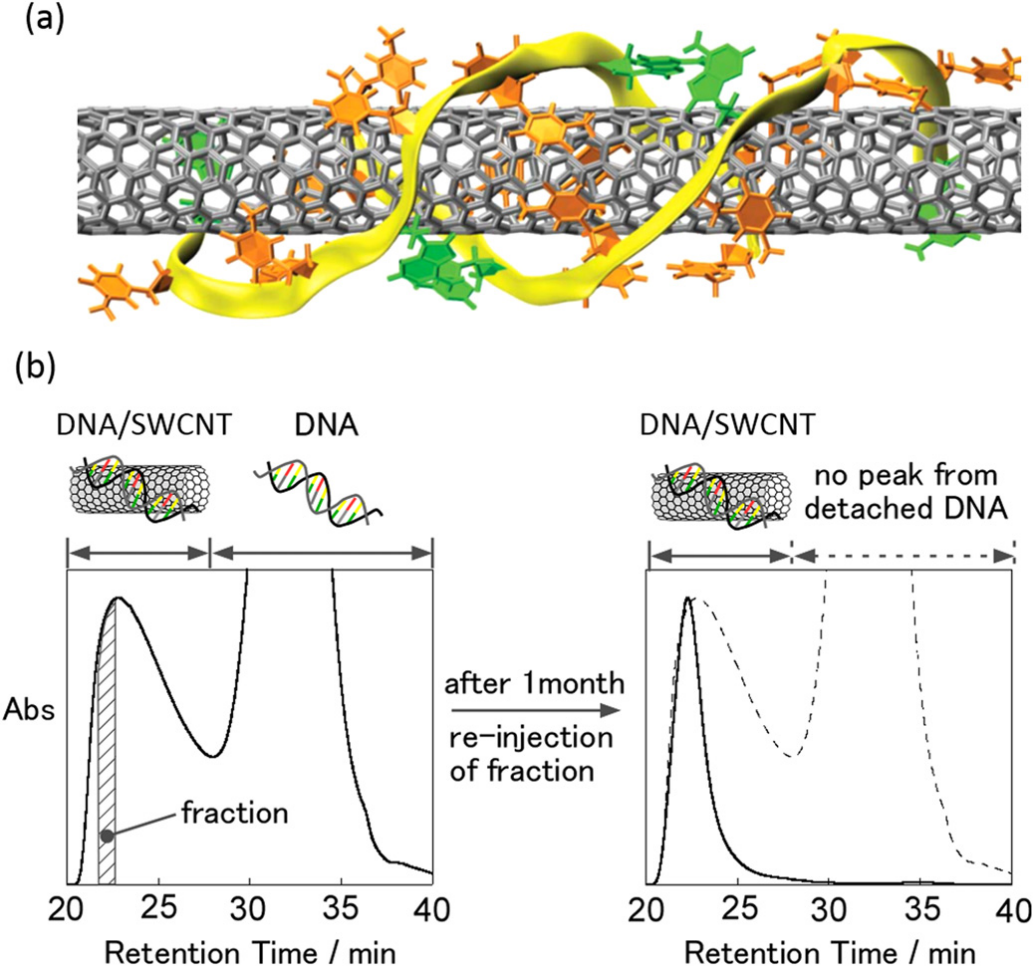
(a) A schematic model of DNA wrapped on SWCNTs. Color coding: orange, thymine; green, adenine; yellow ribbons, backbones. (b) Size exclusion chromatograms of SWCNTs dispersed by dsDNA (left) and the fraction re-injected after 1 month of the separation (right). The chromatograms were measured at 260 nm [55]. Part (a) reprinted by permission from Macmillan Publishers Ltd: X Tu *et al* 2009 *Nature* **460** 250, copyright 2009.

Individual wrapping of SWCNTs using double-strand DNA (dsDNA) and single-strand DNA (ssDNA) in an aqueous system was reported by our group [204] and Zheng *et al* [205], respectively. In these cases, the aromatic nucleic-acid base in DNA are also regarded as the pendant moiety used for the wrapping of CNTs [206–209] in which the stacking of the nucleic-acid base on the SWCNT surfaces leaving highly charged phosphate backbones exposed to water has been proposed both experimentally [206–208, 210] and theoretically (figure 17(a)) [209, 211]. As a matter of fact, dissolution of the CNTs is highly sequence-dependent, and poly-d(T) and d(GT)_10-45_ provides the highest concentration of individual SWCNT aqueous solutions [205, 212]. It was reported that the dispersion efficiency is so high that it exhibits a lyotropic LC phase in the high concentration region [213]. The thermodynamic stability of the DNA wrapping on the CNTs was proved by our group using the gel permeation chromatography (GPC) technique. After removing the unbound DNA in a solution by the GPC technique, no peak attributed to the free DNA was detected even after 1 month, obviously indicating the absence of the detachment of the DNA from the CNT surfaces (figure 17(b)) [55]. Due to the unique combination between biological materials and nano-carbon materials, together with the stable wrapping, a wide range of studies have been carried out for the DNA-wrapped CNTs, such as the conformation transition monitoring of DNA [214], redox sensing of glucose and hydrogen peroxide [215], hybridization detection between ssDNA and their complimentary DNA [56] and uptake estimation of DNA/SWCNTs into a cell [216, 217].

**Figure 18.**
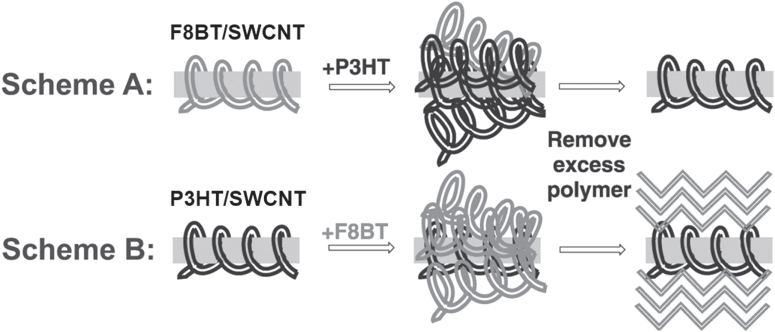
Scheme A shows excess P3HT being added to F8BT/SWCNT. Three days after the addition of excess polymer, any unbound polymer is removed to give P3HT/SWCNT. In Scheme B, the addition of F8BT in P3HT/SWCNT gave P3HT/SWCNT. Reproduced with permission from S D Stranks *et al* 2013 *Adv. Mater.* **25** 4365. Copyright 2013 John Wiley and Sons.

It is important to note that the degree of the stability depends on the polymer structure; thereby, in some cases, exchange of the wrapped polymer with the other polymer dispersant is possible. Sprafke *et al* demonstrated the correlation between the length of the porphyrin oligomers and the binding strength and found that the longer oligomers are able to replace the shorter ones and that the replacement was attributed to an increasing binding affinity with the oligomer length [219]. Chen *et al* demonstrated competitive binding between PFO and PFO-BT and found that the SWCNTs were preferentially functionalized by PFO-BT [220]. In 2013, Stranks *et al* found that PFO-BT was replaced by P3HT in the solution (figure 18) [58]. This fact also suggests that the polymer wrapping can be realized also by the exchange of the surfactant-dispersed CNTs. Indeed, Jeng *et al* reported the wrapping of DNA by adding DNA to the solution of SC dispersed SWCNTs [56]. Such an exchanging procedure is advantageous to avoid damage of the polymers by the dispersion process, such as sonication.

## 3. Characterizations of polymer wrapping

The homogeneous dispersion of the CNTs using polymers as the dispersants is clear evidence of the wrapping of the polymer onto the surfaces of CNTs. However, analysis of further information, such as wrapping geometry, composition ratio and degree of interaction requires detailed characterizations.

The wrapping geometry can be visualized by microscopy techniques such as transmission electron microscopy (TEM), scanning electron microscopy (SEM) and atomic force microscopy (AFM). For instance, helical wrapping of the DNA on CNTs has been extensively studied, especially by TEM [221, 222] and AFM [223–226]. In the case of the PBI wrapping of the CNTs, we successfully visualized the coating structures by SEM based on the difference in the efficiency of the electron scattering from the polymer and CNTs in which non-coated bare islands were also observed [227]. However, since only a limited area is observed in these techniques, and the sample structure sometimes depends on the preparation conditions of the specimen, these microscopic observations need careful interpretation. Generally speaking, it is highly recommended that some spectroscopic evidence, such as absorption or fluorescent spectroscopy, is required to support the microscopic information.

**Figure 19.**
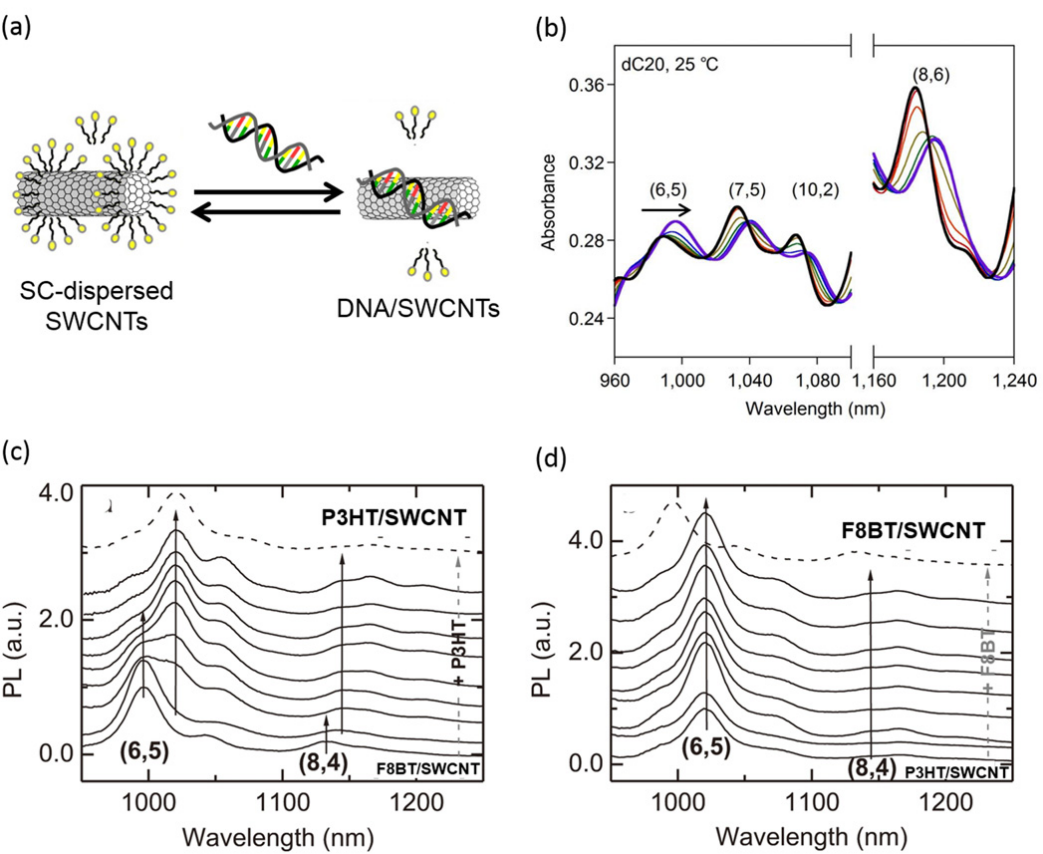
(a) A schematic drawing of the exchange reaction between SC-dissolved SWCNTs (left) and ssDNA/SWCNTs (right). (b) Absorption spectra of the SWCNT in the mixed solution of SC containing ssDNA of 0 (black), 0.0625 (red), 0.156 (orange), 0.313 (yellow), 0.469 (green), 0.938 (blue) and 15.6 *μ*M (purple) at 25 °C. Isosbestic points were observed in the spectral changes. (c), (d) PL spectra of (c) F8BT/SWCNT and (d) P3HT/SWCNT in a chloroform solution excited at 580 nm with an increasing amount of excess (c) P3HT and (d) F8BT and measured 10 days after the addition of excess polymer. Part (b) reprinted by permission from Macmillan Publishers Ltd: Y Kato *et al* 2012 *Sci. Rep.* **2** 733, copyright 2012. Parts (c) and (d) reproduced with permission from S D Stranks *et al* 2013 *Adv. Mater.* **25** 4365. Copyright 2013 John Wiley and Sons.

Absorption spectroscopy, especially in the NIR region, provides useful information, including the dispersion degree of the SWCNTs [228], the degree of wrapping [56] and the replacement of the dispersants [58, 229], since SWCNTs act as the pigment that is sensitive to the surrounding environment. We found that the addition of ssDNA to a SC-dispersed SWCNT solution led to the clear shifts in the absorption spectra in the NIR region with an isosbestic point due to the thermodynamic exchange from SC to ssDNA (figure 19). This finding allowed us to estimate the *ΔT* and *ΔS* values involved in the exchange reaction [229].

In Raman spectroscopy, the wrapped polymers often lead to a shift in the D∗ band, the second-order overtone of the D band that is sensitive to the strain or stress applied to the CNTs from the surrounding media [230–233]. Indeed, the wrapping of PBI on the SWCNTs resulted in the upshifting of the spectrum by 16 cm^−1^ [119]. When the electronic interaction was present, the G-band shift and/or the RBM shift were often observed due to the softening or hardening of the tubes [234].

Fluorescence measurement of the polymer provides strong evidence for the wrapping since an effective energy or electron transfer from the wrapped polymer to the CNTs leads to fluorescence quenching [235–237] when the unbound fluorophore in the bulk solution is successfully removed. In the case of the electron transfer, not only metallic CNTs but also s-SWCNTs can serve as the fluorescent quencher when the LUMO of the s-SWCNTs is lower than the LUMO of the fluorophore [238]. Tezuka *et al* prepared P3HT-wrapped SWCNTs without containing the unbound P3HT and revealed that the short-lived singlet excited state relaxes to yield the exciplex state with the SWCNTs and then rapidly decays to the ground state [239]. On the other hand, not only the excited-state interaction but also the ground-state interaction provides useful information. When SWCNTs are individually dispersed, the peak shifts of the SWCNT fluorescence are observed due to the energy stabilization through the interaction between the polymer and SWCNTs [240].

**Figure 20.**
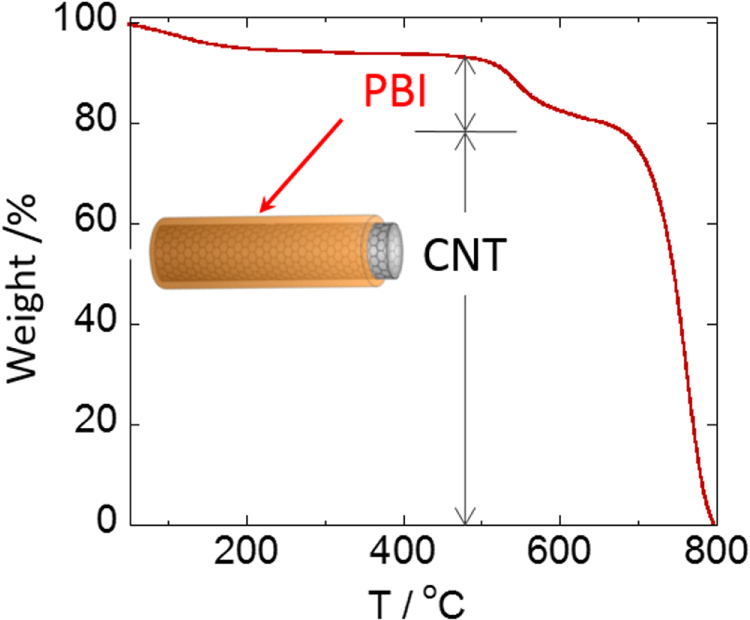
TGA curve of the PBI/CNT.

Characterizations of the polymer-wrapped CNTs after vigorous washing to remove unbound polymer by filtration support information on the average structures. Based on the thermogravimetric analysis (TGA), it is possible to evaluate the composition ratios between the wrapped-polymers and CNTs [59]. For instance, the PBI-wrapped CNTs exhibit a two-step weight reduction at around 520 °C and 700 °C, corresponding to the thermal degradation of the wrapped PBI and CNTs, respectively, and the composition ratio was successfully estimated (figure 20) [59].

## 4. Functions of polymer-wrapped CNTs

**Figure 21.**
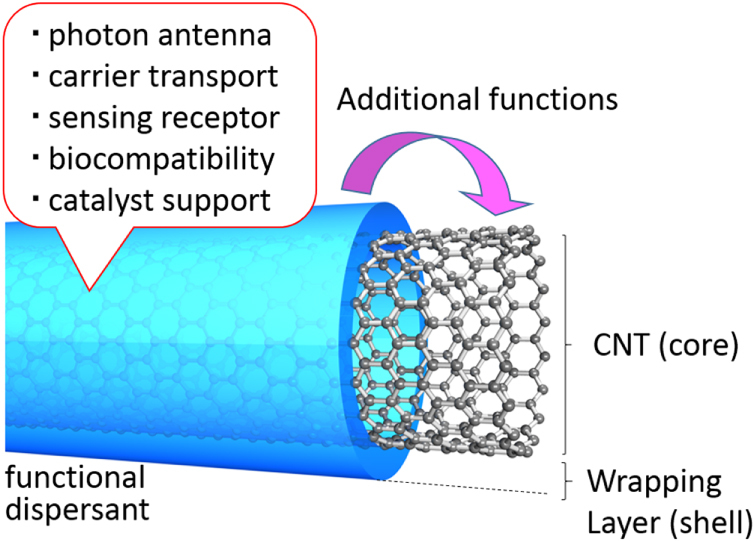
Concept and the role of the functional dispersant.

One of the advantages of polymer wrapping is the synergetic functionalization of CNTs by combining the functions of the polymers. For this target, the strategic design of polymers for wrapping is required. When the wrapped polymer possesses a sufficient affinity to the surfaces of the CNTs, the polymers can remain wrapped even after the vigorous removal of the unbound polymers. As a result, the CNT surfaces are subjected to decoration by the polymer to provide a core–shell structure with an extremely thin polymer layer (figure 21).

### 4.1. Polymer composites

One of the most promising applications of the CNTs is polymer composites in which the CNTs are embedded in the polymer matrices as a filler material to improve the electron conductivity or mechanical strength of the matrices. In order to realize the effective reinforcement using CNTs, a homogeneous dispersion is always the primary requirement [25–30]. To prepare the composite films with a homogeneous dispersion of the CNTs, several approaches were proposed, including (1) melt mixing, (2) *in situ* polymerization and (3) solution blending using either pristine or chemically-modified (oxidized or grafted) CNTs [25–30]. For this purpose, CNTs wrapped by polymers may also serve to increase the miscibility of the matrix polymers. Yan *et al* reported the preparation of a PS-based composite using SWCNTs wrapped by a pyrene-terminated PS in a chloroform solution and found an improvement in the dispersion efficiency compared to the composites prepared in the absence of the pyrene-terminated PS [241]. In this approach, unbound dispersant also remained in the solution, and such a residual possibly contaminates the matrix, depending on the amount of the unbound polymers. On the other hand, if the unbound polymer is removed prior to the composition, well-defined composites can be prepared with a minimum side effect.

**Figure 22.**
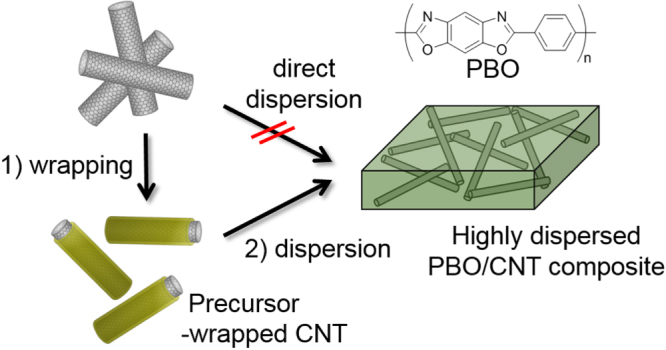
Schematic illustration showing the advantage of the polymer-wrapped CNTs for the preparation of a PBO/CNT composite [120].

In our group, MWCNTs wrapped by a newly developed polybenzoxazole (PBO) precursor (polyhydroxyphenyl amide) was used to blend with the PBO precursor [120]. This two-step blending (figure 22) showed a better homogeneity than the direct blending in the solution since the pristine MWCNTs were hard to disperse, especially in the highly concentrated solution used for film preparation, which is typically over 10 wt% due to their high viscosity. On the other hand, we found that the polymer-wrapped MWCNTs can disperse even in a highly viscous polymer solution without applying any significant shear force, such as sonication, due to the good miscibility of the matrix with the wrapping polymer. Since this approach required a milder condition compared to the reinforcement with the oxidized CNTs involving the severe cutting of the CNTs, effective reinforcements are expected.

### 4.2. Photovoltaic and optoelectronic applications

**Figure 23.**
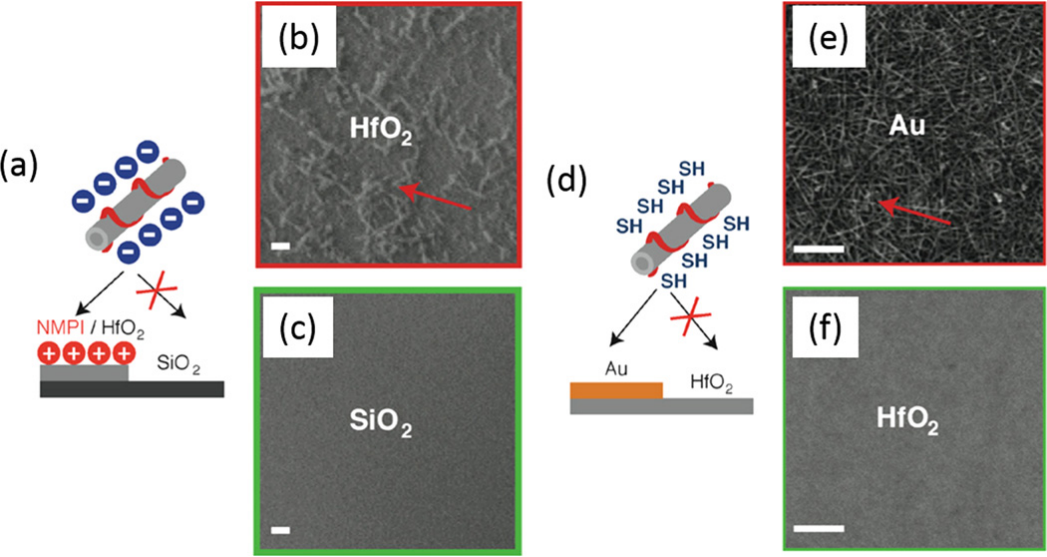
(a) Selective deposition of CNTs based on electrostatic directed assembly. (b), (c) SEM images of (b) HfO_2_ and the (c) SiO_2_ area of the patterned substrate. Scale bars = 1 *μ*m. (d) Selective deposition of CNT based on the metal-ligand directed assembly. (e), (f) SEM images of (e) Au and the (f) HfO_2_ area of the patterned substrate. Scale bars = 100 nm. Reprinted with permission from J M Lobez and A Afzali 2013 *Chem. Mater.* **25** 3662. Copyright 2013 American Chemical Society.

When the wrapping polymers act as the pigment, light-harvesting systems using the CNTs as an acceptor can be fabricated [242]. The unique charge transport features of the CNTs provide an efficient percolation network with a highly efficient exciton dissociation in polymeric bulk heterojunctions with the polymer acting as the donor and the CNT as the acceptor. Based on this concept, poly(3-alkylthiophenes) have been widely used [110, 239]. In 2005, poly(3-octylthiophene) (P3OT) was reported to wrap the SWCNTs and improved the photovoltaic behavior by the photo-induced electron transfer at the P3OT/SWCNT interfaces [243]. The combination of poly(3-alkylthiophenes) and s-SWCNTs forms a heterojunction with a type-II band alignment in which the HOMO and LUMO of the donor are higher than those of the acceptor (figure 23) [244]. This band structure leads to exciton dissociation at the interface with an ultrafast charge transfer from the P3HT to the s-SWCNTs [244–247], and the s-SWCNTs can act as efficient acceptors at the interface [244]. The time-resolved microwave conductivity measurements revealed that the photoexcitation of P3HT results in long-lived carriers due to the efficient spatial separation at the SWCNT–P3HT interface, which readily move electrons away to avoid recombination [248]. Another advantage is that organization of P3HT induced by SWCNTs [249] leads to the improvement of the exciton diffusion as well as the charge mobility in a P3HT/SWCNT composite [250]. It is important to note that the key progress in this field is the developments of the extraction technology of the s-SWCNTs, such as DGU [251] and PFO wrapping [88], since the contamination of m-SWCNTs in the active layer have a detrimental impact on the photocurrent [245], and theoretical studies have predicted that the band alignment of the P3HT/m-SWCNT is unfavorable for charge separation [244]. By utilizing s-SWCNTs, Dabera *et al* revealed the ground-state electron transfer from s-SWCNT to P3HT in the P3HT-wrapped s-SWCNTs, which facilitated the hole transportation property of the s-SWCNTs. The hybrid was used as hole transport layers for bulk heterojunction organic photovoltaics, and the device showed the highest power conversion efficiency (PCE = 7.6%) [252].

Nicholas *et al* developed a unique polymer-wrapping system; namely two different semiconducting polymers, P3HT and PFO-BT, are sequentially wrapped SWCNTs [247]. Due to the difference in the band levels, when the PFO-BT is the inner layer, it acts as a barrier layer, preventing holes created in the P3HT from recombining with electrons in the SWCNTs (figure 23(b)). By incorporating an excess P3HT in order to allow the hole to move away from the interface [58, 247], such structures could be integrated into OPVs, enabling the SWCNTs to be used as electron transporters [253].

### 4.3. Sensors

The high mobility of charge carriers in s-SWCNTs coupled with their high surface area makes them ideal candidates for sensor applications [254]. When the wrapping polymer possesses a stronger affinity for the target analytes, highly selective amperometric sensors can be fabricated [255]. Staii *et al* used s-SWCNTs wrapped by ssDNA in which the DNA sequences were chosen to have a specific binding affinity for a series of analytes, including methanol, propionic acid, trimethylamine (TMA), dinitrotoluene (DNT) and dimethyl methylphosphonate (DMMP) [256]. The introduction of ssDNA significantly enhanced the current signal to as high as 20–30% versus 0.1% for the bare control CNT device with the same exposure dose [257]. Pang *et al* used a cationic polythiophene as the wrapping polymer, providing the binding sites for glucose oxidase and achieving a highly sensitive amperometric biosensor for glucose [258]. Polymer-wrapped CNTs can provide a better biocompatibility compared to the CNTs having a bare surface. For example, higher cell viability was reported for the amylose-wrapped SWCNTs than for the non-wrapped SWCNTs [259], which is advantageous for biosensing applications. In addition, wrapped polymers were also used to incorporate the receptors for sensing. One of the examples is the incorporation of a trinitrotoluene (TNT)-binding peptide into the wrapping layer for the TNT sensing [260]. Tam and Hieu proposed an incorporation of an antibody via electropolymerization of pyrrole in the presence of the antibody, resulting in the formation of PPy-wrapped CNTs containing an antibody [261]. The hybrid functioned as an immune sensor to explore the interactions between the antibody and antigen based on conductivity measurements.

Not only biosensors but also gas sensing are promising applications of polymer-wrapped CNTs. Wang *et al* wrapped CNTs by a hexafluoroisopropanol functionalized thiophene (HFIP-PT). In this hybrid, the HFIP unit was chosen due to the strong hydrogen binding with phosphate esters, which are common structures in many chemical warfare agents, including sarin gas [262]. A FET-based sensor employing polyethyleneimine (PEI)-wrapped CNTs was demonstrated by Qi *et al* and was highly sensitive to NO_2_, while the Nafion-coated FET was selective to NH_3_ [263]. Lobez and Afzali proposed the use of side-chain functionalized polymers for the selective deposition on the patterned substrate in which the selectivity was obtained based on the electrostatic interactions or metal-ligand interaction between the surfaces of CNTs and the substrates (figure 23) [264]. Thiophene-based polymers having phosphonic acid and thiol groups were used to deposit onto the HfO_2_ and Au surface selectively, respectively.

### 4.4. Biotechnology

**Figure 24.**
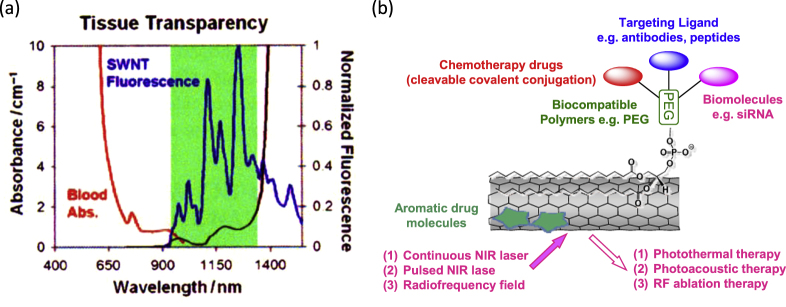
(a) SWCNT fluoresce (blue) in the NIR region. Blood (red) and water (black) absorbance occurs in the visible and NIR region, respectively. The gap in tissue absorbance, which occurs in the NIR region, ensures minimal tissue interference with SWCNT PL emission. (b) A schematic drawing showing various approaches for CNT-based drug delivery and cancer therapies based on PEG-PL as the platform. Part (a) reproduced with permission from A A Boghossian *et al* 2011 *Chem. Sus. Chem.* **4** 848. Copyright 2011 John Wiley and Sons. Part (b) reproduced based on [300].

CNTs also attract much attention in the field of biotechnology [265], including biomedicine [266], biomedical imaging [267], biomedical engineering [268], tissue engineering [269], neurobiology [270], drug discovery [271], drug delivery [272], cancer therapy [273], gene therapy [274] and cell therapy [275] due to their characteristic nano-size as well as their unique optical properties showing a strong light absorption and emission in the NIR region [276]. NIR light is quite useful since most of the components in the body are transparent in the NIR region (figure 24(a)); thus, it is possible to monitor and irradiate CNTs from the outside of the body [276].

However, many toxicological screenings for CNTs revealed the potential risks of the toxicity of CNTs mainly due to the asbestos-like shape of the CNTs [277–279]. Although the degree of toxicity varies from report to report, probably originating from the difference in the length, diameter, metal impurity, dose, type of CNTs, etc [280–283], it is widely recognized that well-functionalized CNTs by biocompatible wrapping exhibit a remarkably reduced toxicity *in vivo* [284–286]. Along this line, one of the most studied molecules for improving long blood circulation of CNTs through a covalent or non-covalent fashion is polyethylene glycol (PEG) [287–293]. The surface coverage with PEG lowers the immunogenicity of the CNTs and prevents their non-specific phagocytosis by the reticuloendothelial system (RES); thereby, their half-life in blood circulation is prolonged [285, 286]. Once the CNTs are stably wrapped with biocompatible materials, they are extremely attractive for biomedicine due to their incredible ability for passing biological barriers across the cytoplasmic and nuclear membrane without generating an immunogenic response [294, 295]. Many researchers have focused on the potential of CNTs for drug delivery, which might be attributed to their exclusive physicochemical features [296, 297].

**Figure 25.**
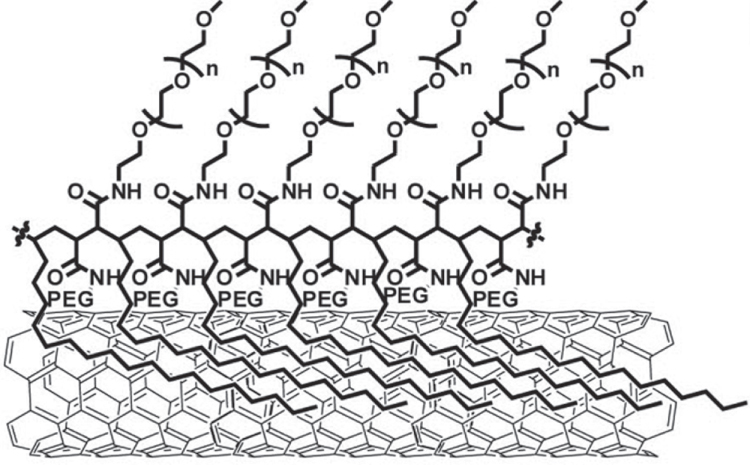
Structure of SWCNTs modified by polymeric PEG. Reprinted with permission from A J Andersen *et al* 2013 *ACS Nano* **7** 1108. Copyright 2013 American Chemical Society.

One of the most successful examples in this field is the series of excellent works explored by Liu *et al* [298, 299]. They wrapped SWCNTs with a PEG-functionalized phospholipid (PEG-PL) and successfully achieved a long blood circulation [285, 300]. By taking advantage of the unique optical properties of non-oxidized SWCNTs, they successfully realized NIR- [301–303] and Raman [304] imaging of the tumor [302, 305] and vessels [303, 306, 307] *in vivo* using PEG-PL/SWCNT. Furthermore, PEG-PL/SWCNT was used as the platform for 1) ligand functionalization for targeting [308], 2) a photo-thermal molecular heater to treat cancer cells and 3) labeling for radio-active imaging (figure 24(b)) [309]. Importantly, SWCNTs wrapped by polymeric PEG synthesized based on the PEGylation of poly(maleic anhydride-alt-1-octadecene) (figure 25) showed a much longer blood circulation half-life than that of PEG-PL/SWCNT due to the pronounced PEG loading [300, 310]. While the packing density of PEG coatings immobilized on PEG-PL/SWCNT with single anchoring points is limited by steric hindrance, polymeric PEG allows continuous binding of the polymer onto the SWCNT surface, yielding a highly dense PEG coating.

**Figure 26.**
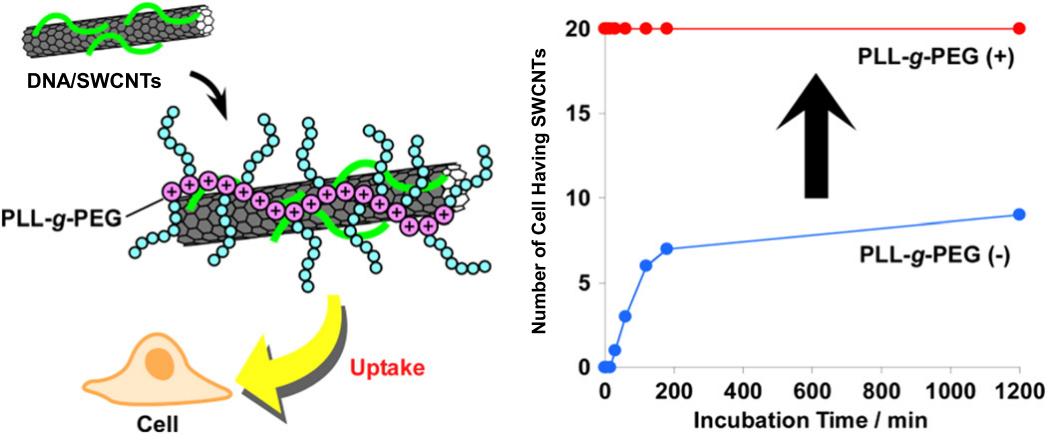
(left) Schematic drawing of PEGylation of DNA/SWCNTs with PLL-*g*-PEG based on the electrostatic interaction. (right) Plots of the number of cells containing SWCNTs as a function of the incubation time. Dramatic enhancement of the cell uptake efficiency is achieved after the PEGylation. Reproduced from T Fujigaya *et al* 2011 *Nanoscale* **3** 4365. Copyright 2011 Royal Society of Chemistry.

In our group, negatively charged ssDNA-wrapped SWCNTs were further hybridized by positively charged poly(L-lysine) grafted by polyethylene glycol (PLL-g-PEG) (figure 26). We found that the obtained ternary hybrid exhibited a dramatic enhancement in the cell uptake efficiency compared to that of the SWCNT wrapped by ssDNA without PLL-g-PEG [57]. In this system, since unbound DNA can be removed prior to the hybridization due to the stable wrapping of ssDNA on SWCNTs, the contamination of the composite without containing SWCNTs was avoided.

### 4.5. Conducting support for the electrocatalyst

**Figure 27.**
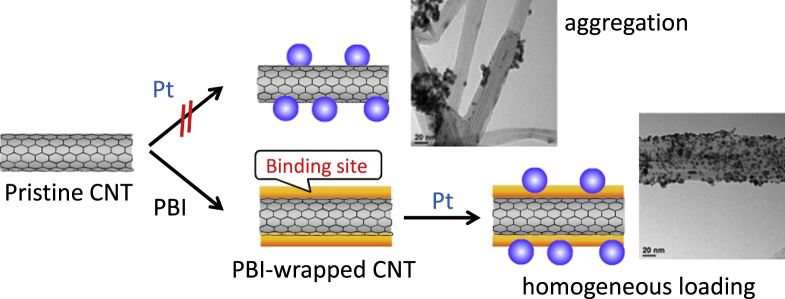
Schematic drawing of the two different approaches for Pt growth, namely the direct growth (upper) and PBI-assisted growth (lower) method.

CNTs are recognized as an ideal supporting material for catalysts used in electrocatalysts, especially for fuel cells due to their higher electrical conductivities [311, 312], lower impurities [313] and higher electrochemical durability [314–318] compared to conventional supporting materials such as carbon blacks (CBs). However, due to the lack of binding sites, such as –COOH and –OH groups, loading of the metal catalyst onto the surfaces of pristine CNTs has been rather difficult. Therefore, the strong oxidation of the CNTs was carried out to introduce the hydrophilic groups [311, 313, 316, 317, 319–325]. However, since the oxidation is known to severely damage the graphitic structure of CNTs and damage the excellent electrochemical stability, a novel methodology to load the catalyst onto the non-oxidized CNTs has been required to utilize the intrinsic electrochemical stability of the pristine CNTs. In this issue, the introduction of binding sites by polymer wrapping of the pristine CNTs offers a promising solution. Until now, CNTs wrapped by poly(allylamine hydrochloride) [326, 327], chitosan [328], PANI [329–331], PDDA [332, 333] and PPy [331, 334, 335] were successfully used to anchor metal nanoparticles onto the surfaces of the pristine CNTs. In our group, PBI-wrapped pristine CNTs were used for the loading of Pt-nanoparticles (figure 27) [59, 60] in which imidazole units in PBI acted as the binding sites for Pt ions through a coordination mechanism, and quantitative loading of the Pt was achieved by a conventional polyol method using an ethylene glycol aqueous solution, H_2_PtCl_6_, as the reducing agent and Pt salt, respectively. The thus-obtained composite (CNT/PBI/Pt) was employed as the electrocatalyst in a polymer electrolyte fuel cell (PEFC) for the first time. As a result of the durability tests using the cells, it was revealed that the fuel cell using PBI-wrapped CNTs showed a remarkable durability compared to the conventional cell using the CB as the supporting material. In this strategy, since PBIs are known to have an excellent proton conductivity after acid doping [336], the wrapping layer of PBI also functioned to fabricate the proton conduction pathway along with CNT networks in the electrocatalyst.

We developed a novel function in the wapping polymer for the purpose of the electrocatalyst application. In this approach, the transformation of the wrapped PBI into a nitrogen-containing (N-doped) graphitic structure by heating in the presence of a metal ion as a graphitization catalyst produced a catalyst layer for the oxygen reduction reaction (ORR) around the CNTs. Recently, N-doped graphitic nanostructures, such as N-doped carbon nanoshells [337], N-doped CNTs [338], N-doped graphite [339, 340] and N-doped graphene [341], have emerged as candidates for the cathode catalyst due to their potential ORR activity. In this application, incorporation of the electron conductivity is one of the key issues for the practical applications. Conversion of the wrapping polymers into the N-doped graphene may facilitate the effective electron delivery into all of the active sites on the surface of CNTs; therefore, a highly active ORR can be expected.

**Figure 28.**
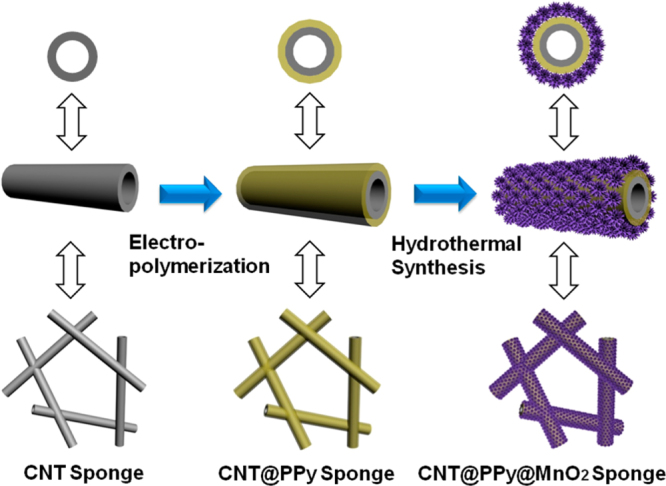
Schematic for the coating of the CNT sponge by PPy and the successive loading of MnO_2_. Reprinted with permission from P Li *et al* 2014 *ACS Appl. Mater. Interfaces* **6** 5228. Copyright 2014 American Chemical Society.

For supercapacitor applications, conducting polymers wrapped on CNTs serve to increase the specific capacitance [342–344]. In these examples, PPy and PANI were often used owing to the ease of wrapping via either chemical or electrochemical polymerization on the surfaces of the CNTs. The recent trend is the additional functionalization with a metal oxide, such as MnO_2_, on the polymer-wrapped CNTs to further increase the specific capacitance [345]. A representative example demonstrated by Li *et al* is the coating of a CNT 3D network (CNT sponge) via electropolymerization of PPy, followed by the MnO_2_ loading by hydrothermal synthesis (figure 28) [346]. Such a ternary system showed the synergetic effect of the components and long cycling life stability [346, 347].

## 5. Summary

We have reviewed polymers that can help to disperse pristine CNTs in solvents via the polymer wrapping mechanism. A wide variety of polymers were found to non-covalently wrap CNTs. Wrapping of CNTs by dispersants based on *π*–*π*, CH–*π* and cation–*π* interactions is a typical mechanism of CNT wrapping. Recently, many polymer dispersants have been developed not only for dispersion of CNTs but also for adding new functions to CNTs. The concept of the functional dispersant is now widely recognized and utilized in many fields, including biotechnology, energy application, etc. The tailorable design of the polymers will further expand the functionality of the polymer-wrapped CNTs, and novel applications using the hybrid are expected.
